# Effect of storage and cooking on the fatty acid profile of omega-3 enriched eggs and pork meat marketed in Belgium

**DOI:** 10.1002/fsn3.197

**Published:** 2014-12-30

**Authors:** Caroline Douny, Rawad El Khoury, Julien Delmelle, François Brose, Guy Degand, Nassim Moula, Frédéric Farnir, Antoine Clinquart, Guy Maghuin-Rogister, Marie-Louise Scippo

**Affiliations:** 1 Department of Food Sciences, Laboratory of Food Analysis, FARAH - Veterinary Public Health, University of LiègeB43bis Bld de Colonster 20, Sart-Tilman, B-4000, Liège, Belgium; 2 Department of Animal Production, Biostatistics, Bioinformatics, Economics and Animal Selection, FARAH – Sustainable animal production, University of LiègeB43bld de Colonster 20, Sart-Tilman, B-4000, Liège, Belgium; 3 Department of Food Sciences, Laboratory of Food Technology, FARAH -Sustainable animal production, University of LiègeB43bis, Bld de Colonster 20, Sart-Tilman, B-4000, Liège, Belgium

**Keywords:** Cooking, egg, fatty acids, GC/MS, pork, shelf-life

## Abstract

The fatty acids (FA) profile was determined in n-3 enriched (Columbus™) Belgian eggs and pork in order to evaluate to what extent the n-3 fatty acids, which are very sensitive to oxidation, are resistant to storage or cooking. In standard eggs or pork, no change of the fatty acid profile was observed after storage or cooking without culinary fat, as well as in Columbus™ eggs and pork after storage. Some cooking processes (eggs in custard and meat in oven) induced a slight significant loss of n-3 fatty acids in Columbus™ eggs or pork (11.1% in fat from eggs cooked in custard vs. 15.3% in raw Columbus™ eggs and 11.0% in fat from oven cooked meat vs. 11.6% in raw Columbus™ meat). As expected, when Columbus™ pork is cooked with culinary fat, its fatty acid profile is modified according to the nature of the fat used.

## Introduction

The essential C18 polyunsatuared fatty acids (PUFA) n-6 linoleic (LA) and n-3 *α*-linolenic (LNA) acids are precursors of long chain C20 and C22 highly unsaturated fatty acids, and compete, in this process of elongation, for the same Δ^6^ desaturase enzymes. So, an excessive intake of n-6 compared to n-3 fatty acids could lead to a deficiency in n-3 eicosapentaenoic acid (EPA) and docosahexaenoic acid (DHA), which are said semi-essential fatty acids. EPA and DHA are well known for their beneficial effects, in particular, to prevent different pathologies, mainly cardiovascular diseases (Delgado-Lista et al. 2012; Lovegrove and Griffin 2013). Nowadays, it is considered that the general western diet contains an excess of n-6, with a ratio between n-6 and n-3 FA around 10–15 (Simopoulos 2008). This imbalance is thought to contribute to the appearance of the “modern day” metabolic syndrome, including health problems such as cardiovascular diseases, type 2 diabetes, obesity, allergies, inflammations, cancers, stress etc. For several years, it was considered that for humans, the ideal ratio between fatty acids n-6 and n-3 in food was 1:1. A recent study realized for the American Heart Association (Harris et al. 2009) showed that n-6 fatty acids have no pro-inflammatory effects in humans. Furthermore, this study confirms the hypo-cholesterolemia potency of n-6 fatty acids and recommends abandoning the idea that n-3 and n-6 fatty acids display opposite effects and to not use anymore the ratio n-6/n-3 to qualify the intake of these fatty acids. Moreover, this study recommends to increase the n-3 and to maintain the n-6 fatty acids dietary intake.

Increasing the amount of n-3 fatty acids in animal feed is a way to increase human intake of those compounds through the consumption of food from animal origin other than fatty fish, the major natural dietary source of long chain n-3 fatty acids. Examples from literature have shown the possibility to obtain n-3 fatty acids enriched meat (Raes et al. 2004; Wood et al. 2004), eggs (Jiang and Sim 1992; Cherian et al. 2007a,b) or dairy products (Oeffner et al. 2013). Subsequently, many products enriched with n-3 fatty acids can be found in the market: meat, milk and dairy products, eggs… For example, in Belgium, a Walloon company developed a patented animal feed (Columbus™ feed) containing 5% of linseed oil (Remacle et al. 2001) suitable for hens that produce Columbus™ eggs with a ratio of omega-6 and omega-3 fatty acids of 1:1. The same strategy was used more recently to produce Columbus™ pork.

Polyunsaturated fatty acids oxidation in food is generally favored by thermal processing as well as storage (Lopez-Bote et al. 1998; Nurnberg et al. 1999; Hayat et al. 2010). Eggs and pork are categories of foods that are eaten cooked, that is, after thermal processing. Consequently, due to the possible oxidation, the quantity of PUFA remaining in the food when it is eaten, that is, after storage and/or cooking, could be lower than the initial content in the raw product.

Our study investigated the impact of storage and cooking on the polyunsaturated fatty acids content of two products rich in omega-3 fatty acids marketed in Belgium: Columbus™ eggs and Columbus™ pork.

## Materials and Methods

### Chemical reagents and cooking ingredients

Free and methylated fatty acids standards were purchased from Sigma-Aldrich (St. Louis, MO). Hexane and toluene were of Picograde quality and provided by Promochem (Wesel, Germany). Methanol and water were of Chromanorm quality and provided by VWR International (West Chester, PA). Hydrochloric acid, 37%, was from Merck (Darmstadt, Germany).

Individual stock solutions of each fatty acid standard in hexane were used to prepare a pool of 23 FA standards, for the external calibration. Nonadecanoic acid (C19:0) was used as internal standard and gadoleic acid methyl ester (C20:1-ME) was used as injection standard.

The certified reference material (CRM) BCR-162R (made of soya-maize oil blend) was purchased at the Institute for Reference Materials and Measurements (IRMM, Geel, Belgium).

Skimmed milk, sugar, flour, corn starch, palm oil, butter, margarine, sesame, and peanut oils were purchased in a local supermarket.

### Fat extraction

Samples were weighed, homogenized (for raw whole eggs) or minced (meat and cooked eggs), and lyophilized for 48 h (Benchtop, Virtis; SP Industries, Warminster, PA). The dry matter was weighed and the water content was then calculated. Then, extraction of the total lipids was done using hexane at 125°C for 20 min in an accelered solvent extraction (ASE) system (ASE 200; Dionex, Sunnyvale, CA) and the fat residue was weighed.

### Preparation of fatty acid methyl esters

Fifty milligrams of fat extracted with ASE were mixed with 5 mL hexane and 10 *μ*L were used for the saponification/methylation of the fatty acids. Internal standard nonadecanoic acid (C19:0) was then added and hexane was evaporated to dryness under a stream of nitrogen. One milliliter toluene and 2 mL sulfuric acid 2% (v/v, in methanol) were added to the fat and the capped tube was heated in a water bath at 100 °C for 1 h, with vigorous agitation thanks to a magnetic stirrer. Then, 3 mL NaCl 5% were added and the methyl esters were extracted with two times 2 mL hexane. The extract was washed with 4 mL K_2_CO_3_ 2% (w/v) and Na_2_SO_4_ was added to a part of the extract. The extract was then evaporated to dryness in a Savant™ Universal SpeedVac™ Vacuum System (Thermo Fisher Scientific, Waltham, MA) in order to eliminate the toluene. Three hundred and fifty-five microliters hexane was added and the tube was vortexed. Finally, 80 *µ*L was transferred into an injection vial and 20 *µ*L gadoleic acid methyl ester (C20:1-ME) was added to be used as the injection standard.

For the calibration curve, the same protocol was applied to hexane solutions containing a pool of 23 fatty acids, at six different concentration levels (from 0.06 to 16.68 ng *μ*L^−1^).

### GC–MS separation, detection, and quantification of fatty acids

The method used to analyze fatty acid methyl esters (FAME) was adapted from Aldai et al. (2006). FAME were separated on a Focus GC gas chromatographer (Thermo Fisher Scientific) using a CP-Sil88 column for FAME (100 m × 0.25 mm, 0.2 *μ*m) (Varian; Agilent Technologies, Santa Clara, CA) and analyzed with an ion trap PolarisQ mass spectrometer (Thermo Fisher Scientific). The GC conditions were: inlet: 250°C; splitless injection; helium as the carrier gas at 1.5 mL min^−1^; temperature program: 55°C for 1 min, followed by an increase of 5°C min^−1^ to 180°C, then 10°C min^−1^ to 200°C for 15 min, then an increase of 10°C min^−1^ to 225°C for 14 min; total run time was 59.50 min. Injection volume was 1 *μ*L. The peaks were identified by comparing their mass spectrum and retention times with those of the corresponding standards. The MS conditions were: transfer line: 250°C; ion source: 220°C; collision energy: 35 eV; positive ionization mode. The FAME were detected using selected ion monitoring (SIM) mode in five segment windows. In each chromatographic run, different ions were monitored for each fatty acid analyzed, which allowed to perform detection and quantitative analysis: m/z 101 + 143 for saturated, 79 + 91 for mono and polyunsaturated fatty acids.

The 23 FAME, the internal standard, and the injection standard were separated in a run time of 1 h using the optimized GC–MS parameters described in Meat samples section.

For quantification, a 6-point calibration curve containing standard solutions and the internal standard was performed for each of the 23 fatty acids methyl esters determined. The response (ratio between fatty acids methyl esters and the internal standard peak areas) was plotted against standard concentrations. A linear regression was used and no “fit weighting” was applied.

### Eggs

Columbus™ (“omega-3 rich”) and standard eggs were bought in a local supermarket and were from barn raised hens (purchase date corresponding to 1 week after laying). For both types, all eggs were of medium size (53–63 g). A total of 252 eggs coming from four different batches were used for two studies: one batch for the study of the impact of the storage conditions and three batches for the study of the impact of cooking.

#### Egg cooking experiment

Eggs were cooked in a water bath (hard-boiled 4, 10, and 15 min), in a pan (scrambled and “au plat”) and in an oven (in Savoy cake and in custard). For each cooking experiment, eggs were kept at room temperature 30 min before cooking. Temperatures in water bath, oven, and eggs were monitored during cooking procedures with temperature probes from Testo (Lenzkirch, Germany), with a measurement uncertainty of 0.5°C.

- Water-bath cooking: Water temperature was set at 100 °C. Hard-boiled eggs (4, 10, and 15 min) were cooled 5 min in ice to stop the cooking.
- Pan cooking: eggs were cooked (during 3 min in each case), scrambled (two eggs), and “au plat” (one egg) without oil, with the use of a polytetrafluoroethylene (PTFE) cooking foil.
- Custard (oven cooking): 250 mL skimmed milk was boiled and mixed with 50 g sugar. Then, two eggs were beaten and added to the milk. The mixture was cooked in the oven set at 180°C for 45 min in a water bath.
- Savoy cake: Three egg yolks were mixed with 50 g sugar then 40 g flour, and 30 g corn starch were added. Three egg whites were beaten firmly and 20 g sugar were added. The beaten whites were then folded into the egg yolk mixture; then the mixture was poured into a cake pan and baked for 30 min in the oven set at 160°C.

All eggs have been weighed before and after cooking. Each cooking experiment used 15 eggs of each type: one raw, three hard-boiled (1 for 4 min, 1 for 10 min, and 1 for 15 min), two pan cooked “au plat” (1 without fat and 1 with fat), four pan cooked scrambled (2 without fat and 2 with fat), two in custard and three in Savoy cake. Each experiment was repeated three times with eggs from different batches, leading to a total of 45 eggs of each type.

#### Egg storage experiment

Raw and hard-boiled (10 and 15 min) Columbus™ and standard eggs were stored 6 weeks in the dark at +4°C and +20°C. Each week, until the sixth week of storage, two eggs were sampled for the fatty acid profile determination. Each condition was in triplicate for day zero and in duplicate for the other storage times. A total of 81 eggs of each type were used for the storage experiment. All eggs from one type were from a single batch.

#### Egg control samples

For each type of eggs, a total of six raw eggs were analyzed to be used as control for both experiments: three raw eggs coming from the cooking experiment (three different batches) and three raw eggs coming from the storage experiment.

### Meat samples

The meat was coming from the shoulder of one Columbus™ pig and one standard pig (both Piétrain), provided by the company Marcel Biron & Fils (Bouffioulx, Belgium). Samples of ground meat of ∽70 g each, coming from two different batches, were used for cooking and storage experiment: one batch for the study of the impact of the storage conditions and one batch for the study of the impact of cooking.

#### Pork cooking experiment

Samples of ground meat (∽70 g) coming from one Columbus™ pig and one standard pig were used for each cooking procedures. A total of 18 samples of each type of meat were used for the cooking experiment.

Oven cooking experiment: the meat (four samples of each category of pig) was cooked in a glass beaker in the oven set at 180°C without culinary fat until the core temperature reached 80°C (between 20 and 22 min). During the oven cooking process, the core temperature was monitored continuously with thermic probes (TC type T; Testo).

Pan-frying experiment: the meat (two samples) was cooked in the pan without fat or with four different culinary fats: butter, margarine, sesame, and peanut oils. The amount of culinary fat used was around 5% of the weight of the raw meat. Each condition was realized in duplicate and pan frying took 20 min, during which the meat was cooked 15 min on one side and 5 min on the other side.

#### Raw pork storage experiment

Different conditions of storage were investigated for raw ground meat samples: vacuum-packed, plastic-bag-packed, and polypropylene tray with plastic wrap. Samples stored in plastic-bag and polypropylene tray with plastic wrap were kept at +4°C and at −20°C, while samples stored under vacuum were kept at +4°C. All samples were stored in the dark and analyzed in duplicate after a determined number of weeks, depending on the package type and temperature: 1 and 2 weeks for plastic bag and plastic tray at +4°C; 2, 4, 6, 8 weeks for plastic bag and plastic tray at −20°C and 3, 4, 6 weeks for under vacuum at +4°C. A total of 33 samples of each type of meat were used for the storage experiment.

#### Meat control samples

For each category of pigs (Columbus™ or standard), a total of seven meat samples were analyzed to be used as control for both experiments: four samples coming from the cooking experiment and three samples coming from the storage experiment.

### Statistical analysis

Statistical Analysis System (SAS Institute, Cary, NC) was used for statistical analysis. Significant differences between treatments were tested using the analysis of the variance (two-way ANOVA) and generalized linear models (GLM) procedure of the SAS software. Post hoc analyses were used to compare conditions and levels of significant effects were compared using least-square means and associated standard error (significant for *P* < 0.05). The least-square mean corresponds to the mean corrected for all other effects in the model.

## Results and Discussion

### Choice of the extraction technique

To extract lipids from the samples, ASE was used, with hexane at high pressure and at a temperature above its boiling point. To our knowledge, no data were found in the literature about the use of ASE using hexane as extraction solvent. Indeed, most of the methods developed with this technique use either chloroform/methanol (2:1, v/v) or isopropanol/hexane (2:3, v/v) to extract lipids from biological matrices such as food (Ruiz-Rodriguez et al. 2010). Nevertheless, many authors propose to extract lipids from food using another technique called microwave Soxhlet extraction, with hexane as extraction solvent (Priego-Capote et al. 2004, 2007; Virot et al. 2007, 2008). The results obtained in this study with ASE using hexane were in good agreement with those obtained with microwave Soxhlet extraction using hexane for major fatty acids identification in eggs or pork.

### Performances of the GC–MS analytical method

The developed method to measure fatty acids fulfills the criteria indicated in the Commission Decision 2002/657/EC (European Parliament and Council Directive No 2002/657/EC 2002/657/EC 2002/657/EC, 2002) which provides guidelines to evaluate the performance of the screening and confirmatory methods used for organic residues and contaminants analysis: retention time and ions ratios, selectivity, specificity, repeatability, reproducibility (data not shown).

A good separation was achieved for the peaks of all compounds except for the methyl esters of C24:0 and C20:5n-3 (EPA). Nevertheless, C24:0 being a saturated fatty acid and C20:5n-3 being a polyunsaturated fatty acid, the problem was solved by quantifying them with different m/z ratios such as 101 + 143 for C24:0 and 79 + 91 for C20:5n-3 (EPA). For calibration curves, the linear regression provided a good curve fitting, that is, with low residue values and correlation coefficients *R*² associated with those curves higher than 0.98 for the 23 fatty acids, and demonstrated the linearity of the dose–response curve within the working range (data not shown).

The limit of quantification (LOQ) was fixed as the content of fatty acids corresponding to the first point of the calibration curve (after checking that the signal to noise ratio was higher than 10 at that level), and corresponds to about 0.1–0.5% of total fatty acids, depending on the standard. The limit of detection (LOD) was set at LOQ/2, after checking that the signal to noise ratio was higher than 3 at that level.

A CRM was used to assess the performance of the developed method. The CRM BCR-162R (soya-maize oil blend) contains assigned percentage of 10.74% of palmitic acid, 2.82% of stearic acid, 25.4% of oleic acid, 54.13% of linoleic acid, and 3.35% of α-linolenic acid. The measured values (*n* = 39) were, respectively, 105.9%, 104.1%, 107.9%, 99.9%, and 103.0% of the certified content (data not shown).

### Eggs

#### Characterization of raw eggs

The average water content and fat contents were, respectively, 75.7 ± 1.5% and 8.8 ± 1.1% for Columbus™ eggs and 76.6 ± 0.9% and 7.5 ± 0.6% for standard eggs (*n* = 6), expressed on fresh weight basis. In both standard and Columbus™ whole raw eggs, the three major fatty acids are, in decreasing order, oleic acid (45.8 vs. 40.7%), palmitic acid (23.7 vs. 19.1%), and linoleic acid (16.3 vs. 14.3%) (Table1). As expected, Columbus™ eggs contained a much higher proportion of n-3 LNA fatty acid (12.7%) than standard eggs (1.1%), and consequently, more PUFA (30.2% in Columbus™ eggs vs. 20.2% in standard eggs) and less SFA and MUFA (26.3% and 43.5%, respectively, in Columbus™ eggs vs. 31.1% and 20.2%, respectively, in standard eggs). Interestingly, the DHA (C22:6, n-3) content (1.4% in Columbus™ eggs vs. 0.7% in standard eggs) is also increased in eggs from hens fed with linseed oil containing diet, while the ARA (C20:4, n-6) content is decreased (0.6% in Columbus™ eggs vs. of 1.4% in standard eggs). The n-6/n-3 ratio was found equal to 7.5 for standard eggs and to 1.0 for Columbus™ eggs, which is a value that meets nutritional recommendations of reducing this value below 5 (AFSSA - Agence Française de Sécurité Sanitaire des Aliments 2003).

**Table 1:** Fatty acid composition (g/100 g) of standard and n-3 enriched (Columbus™) raw eggs

	Fatty acid content	
	Columbus™ egg (*n* = 6)	Standard egg (*n* = 6)
10:0	ND	ND
12:0	ND	ND
13:0	ND	ND
14:0	0.5 ± 0.1	0.5 ± 0.1
16:0	**19.1 ± 0.4**	**24.2 ± 1.3**
17:0	ND	ND
18:0	6.9 ± 0.2	6.6 ± 0.5
20:0	ND	ND
22:0	ND	ND
24:0	ND	ND
16:1 (n-7)	2.7 ± 0.3	2.9 ± 0.7
17:1 (n-7)	ND	ND
18:1 (n-9)	**40.7 ± 1.8**	**43.9 ± 4.1**
18:2 (n-6) (LA)	14.2 ± 0.8	17.9 ± 4.7
20:2 (n-6)	ND	ND
18:3 (n-3) (LNA)	**12.7 ± 2.0**	**1.2 ± 0.4**
18:3 (n-6)	ND	ND
20:3 (n-3)	ND	ND
18:4 (n-3)	ND	ND
20:4 (n-6) (ARA)	**0.6 ± 0.1**	**1.4 ± 0.1**
20:5 (n-3) (EPA)	**0.6 ± 0.1**	**0.3 ± 0.04**
22:5 (n-3) (DPA)	**0.6 ± 0.1**	**0.3 ± 0.1**
22:6 (n-3) (DHA)	**1.4 ± 0.1**	**0.8 ± 0.2**
Σ SFA	**26.5 ± 0.7**	**31.3 ± 1.4**
Σ MUFA	**43.4 ± 1.7**	**46.8 ± 4.5**
Σ PUFA	**30.1 ± 2.0**	**21.9 ± 5.3**
n-6 fatty acids	14.8 ± 0.8	19.3 ± 4.8
n-3 fatty acids	**15.3 ± 2.0**	**2.6 ± 0.6**
n-6/n-3	**1.0 ± 0.2**	**7.5 ± 0.8**

Mean ± standard deviation (SD) of six eggs from four different batches: three eggs coming from the storage experiment (one single batch) and three eggs coming from the cooking experiment (three different batches). Significant differences comparing Columbus™ and standard eggs are indicated in bold (*P* < 0.05). SFA, saturated fatty acids; MUFA, monounsaturated fatty acids; PUFA, polyunsaturated fatty acids; ND, not detected.

The proportion of 12.7% of α-linolenic (LNA) acid in the Columbus™ eggs appeared to be higher than the values (between 3.4% and 10.7%) reported in the literature for eggs from hens fed with diet containing linseed products (Meynier et al. 2014; Galobart et al.*,* 2001; Baucells et al. 2000; Halle and Schöne 2013; Ferrier et al. 1995; Ren et al. 2013; Botsoglou et al. 2012a,b,c). The same authors reported for the same eggs a DHA content varying from 1.5% to 2.3% versus 1.4% in Columbus eggs, while a proportion of 3.2% or 5.8% of DHA can be reached when hens are fed with fish oil containing feed (reported in, respectively, Baucells et al. 2000 and Botsoglou et al. 2012a). Another author reported 4.5% of LNA and 2.1% of DHA in commercial omega-3 eggs in Australia (Samman et al. 2009).

Columbus™ eggs can be considered as “high omega-3 fatty acids” products, according to the Regulation (EC) no. 1924/2006 (European Parliament and Council Directive No 1924/200620062006, 2006), as they contain more than 0.6 g LNA per 100 g (based on 7.5% fat containing 12% LNA, they contain about 0.9 g of LNA per 100 g).

#### Egg cooking experiment

##### Evolution of the core temperature during egg cooking

The evolution of the core temperatures during each cooking experiment was monitored with Testo probes. Temperature values are expressed as mean ± standard deviation of three independent cooking experiments. In the different cooking procedures where the temperature was monitored, the maximum core temperatures recorded at the end of the cooking time in the standard eggs were 118.3 ± 4.3°C, 102.6 ± 15.6°C, 49.3 ± 2.0°C, 71.9 ± 5.2°C, and 80.5 ± 7.0°C for Savoy cake, custard, and hard-boiled eggs cooked 4, 10, and 15 min, respectively. In Columbus™ eggs, the maximum core temperatures recorded were 97.4 ± 4.3°C, 88.7 ± 2.1°C, 37.4 ± 4.5°C, 65.5 ± 5.8°C, and 77.4 ± 4.9°C for Savoy cake, custard, and hard-boiled eggs cooked 4, 10, and 15 min, respectively. Surprisingly, the core temperatures recorded for Columbus™ eggs were significantly lower than for standard eggs in the Savoy cake during the last 10 min of cooking.

##### Fatty acid profile of eggs before and after cooking

Table2 shows the results obtained for the sum of SFA, MUFA, PUFA, n-3, and n-6 fatty acids, and the LNA (C18:3 n-3), DHA (C22:6 n-3), and ARA (C20:4 n-6) proportion, in raw and cooked eggs. The n-6/n-3 ratio has been calculated as well.

**Table 2:** Effect of cooking on the fatty acid composition (g/100 g) of standard and Columbus™ eggs

Egg type	Condition	Σ SFA		Σ MUFA		Σ PUFA		n-3		n-6		C18:3(n-3) LNA		C22:6(n-3) DHA		C20:4(n-6) ARA		n-6/n-3	
		LSM	SE	LSM	SE	LSM	SE	LSM	SE	LSM	SE	LSM	SE	LSM	SE	LSM	SE	LSM	SE
Standard	Raw	31.3	0.8	46.8	2.2	21.9	2.9	2.6	0.3	19.3	2.6	1.2	0.2	0.8	0.1	1.4	0.1	7.5	0.8
	HB 4 min	31.1	0.8	47.4	2.2	21.5	2.9	2.4	0.3	19.1	2.6	1.2	0.2	0.7	0.1	1.3	0.1	8.2	0.8
	HB 10 min	32.6	0.8	44.5	2.2	22.8	2.9	2.6	0.3	20.3	2.6	1.2	0.2	0.8	0.1	1.6	0.1	7.9	0.8
	HB 15 min	31.8	0.8	44.9	2.2	23.4	2.9	2.6	0.3	20.8	2.6	1.2	0.2	0.8	0.1	1.5	0.1	8.2	0.8
	“Au plat”	31.7	0.9	46.8	2.5	21.5	3.3	2.4	0.4	19.1	3.0	1.1	0.2	0.7	0.1	1.5	0.1	8.2	0.9
	Scrambled	32.2	0.9	45.4	2.5	22.3	3.3	2.4	0.4	19.9	3.0	1.2	0.2	0.8	0.1	1.5	0.1	8.3	0.9
	Custard	32.6	1.1	46.0	3.1	21.4	4.1	2.9	0.5	18.5	3.7	1.3	0.2	0.6	0.2	1.1	0.1	6.3	1.1
	Savoy cake	32.1	0.9	45.3	2.5	22.6	3.3	2.5	0.4	20.1	3.0	1.3	0.2	0.7	0.1	1.3	0.1	8.2	0.9
Columbus™	Raw	26.5	1.0	43.4	1.2	30.1	1.8	15.3	1.5	14.8	0.5	12.7	1.6	1.4	0.1	0.6	0.1	1.0	0.1
	HB 4 min	25.5	1.0	42.2	1.2	32.4	1.8	16.9	1.5	15.5	0.5	14.3	1.6	1.5	0.1	0.7	0.1	0.9	0.1
	HB 10 min	27.2	1.0	43.3	1.2	29.5	1.8	14.8	1.5	14.7	0.5	12.4	1.6	1.3	0.1	0.6	0.1	1.0	0.1
	HB 15 min	28.2	1.0	43.1	1.2	28.7	1.8	13.7	1.5	15.0	0.5	11.2	1.6	1.4	0.1	0.7	0.1	1.1	0.1
	“Au plat”	27.0	1.1	41.4	1.4	31.7	2.0	16.2	1.7	15.5	0.5	13.6	1.9	1.4	0.1	0.6	0.1	1.0	0.1
	Scrambled	28.0	1.1	42.2	1.4	29.8	2.0	14.6	1.7	15.3	0.5	12.0	1.9	1.5	0.1	0.6	0.1	1.1	0.1
	Custard	31.7*	1.1	42.6	1.4	25.8*	2.0	11.1*	1.7	14.6	0.5	8.8*	1.9	0.9*	0.1	0.5	0.1	1.4*	0.1
	Savoy cake	28.9*	1.1	42.4	1.4	28.7	2.0	13.6	1.7	15.1	0.5	11.3	1.9	1.3	0.1	0.5	0.1	1.1	0.1

Least-square means (LSM) and Standard errors (SE) of six eggs from four different batches for raw and hard-boiled eggs, two eggs from two different batches for standard custard, and three eggs from three different batches for each other cooking condition. Significant differences comparing to the raw SFA, saturated fatty acids; MUFA, monounsaturated fatty acids; PUFA, polyunsaturated fatty acids; n-3, n-3 fatty acids; n-6, n-6 fatty acids; LNA, *α*-linolenic acid; DHA, docosahexaenoic acid; ARA, arachidonic acid; HB, hard-boiled eggs condition are indicated by asterisks (^*^) and in bold (*P* < 0.05).

Results are expressed in percent of total identified fatty acids, as least-square means and standard errors of six eggs from four different batches for raw and hard-boiled eggs and three eggs from three different batches for each other cooking condition. The statistical analysis applied to the data corrected the heterogeneity observed between batches.

The fatty acids composition of the standard eggs was not significantly influenced by any cooking procedure. Indeed, the SFA, MUFA, and PUFA, as well as the n-3 and n-6 fatty acids contents showed no significant difference between raw and cooked eggs. The LNA, DHA, and ARA contents were neither influenced (Table2). Murcia et al. (1999) studied the effect of cooking on the fatty acid profile of standard eggs (containing 36.5% of C18:1, 29.2% of C16:0, and 26.2% of C18:2). Concerning the PUFA, they reported a decrease of C18:2 (LA), C18:3 (LNA), and C20:4 (ARA) in scrambled standard eggs (omelette) and microwaved eggs compared to raw eggs. For boiled standard eggs, they observed an increase of LA and ARA after boiling 3 min and a decrease of LA and ARA after boiling 10 min, while LNA was constant in both boiling conditions. No information concerning the statistical significance of the observed results was provided.

For Columbus™ eggs, hard-boiling, scrambling, or “au plat” cooking of had no significant effect on PUFA and n-3 fatty acids contents, compared to raw eggs, while significant effects of the cooking were observed in custard and Savoy cake (Table2). In custard, the percentage of LNA and DHA in the total fatty acids decreased to 8.8% and 0.9%, respectively (it was 12.7% and 1.4%, respectively, in raw eggs), with a subsequent decrease of the percentage of PUFA (25.8%) and an increase of the percentage of SFA (31.7%) (in raw eggs, PUFA and SFA were 30.1% and 26.5%, respectively). These changes resulted in an increase of 40% of the n-6/n-3 ratio (1.4 instead of 1.0 in raw eggs). In Savoy cake, the only significant difference, compared to raw eggs, was recorded for the SFA content, but to a lesser extent than in custard (28.9% and 31.7% in Savoy cake and custard, respectively, vs. 26.5% in raw eggs). This increase of SFA in Savoy cake probably comes from the slight, apparently not significant, decrease of n-3 content (15.3% in raw eggs vs. 13.6% in Savoy cake). The decrease in PUFA observed in Savoy cake and custard prepared with ColumbusTM eggs can be explained by the temperatures reached in the core of the egg preparation (97.4 ± 4.3°C and 88.7 ± 2.1°C, respectively), which are the highest temperatures recorded among the different cooking experiments (see Evolution of the core temperature during egg cooking). The fact that such a decrease is not observed in standard eggs can be explained by the 10 times higher content of ColumbusTM eggs in LNA, a polyunsaturated fatty acid displaying three double bonds, thus much more sensitive to oxidation that the two double bonds LA contributing the most to the PUFA content of standard eggs.

Other studies reported that boiling or scrambling omega-3 enriched eggs, slightly decreased the C18:3 (LNA) and C22:6 (DHA) content (LNA: 7.41% and 7.28% in boiled and scrambled eggs, respectively, vs. 7.84% in raw eggs; DHA: 1.10% and 1.02% in boiled and scrambled eggs, respectively, vs. 1.62% in raw eggs) (Botsoglou et al. 2012c), while Cortinas et al. (2003) showed a significant decrease for DHA (and not LNA) in eggs coming from hens fed with fish oil containing feed, after scrambling only (and not boiling). Van Elswyk et al. (1992) reported that cooking (boiling or scrambling) did not alter the fatty acid composition of omega-3 enriched eggs.

#### Egg storage experiment

**Figure 1:**
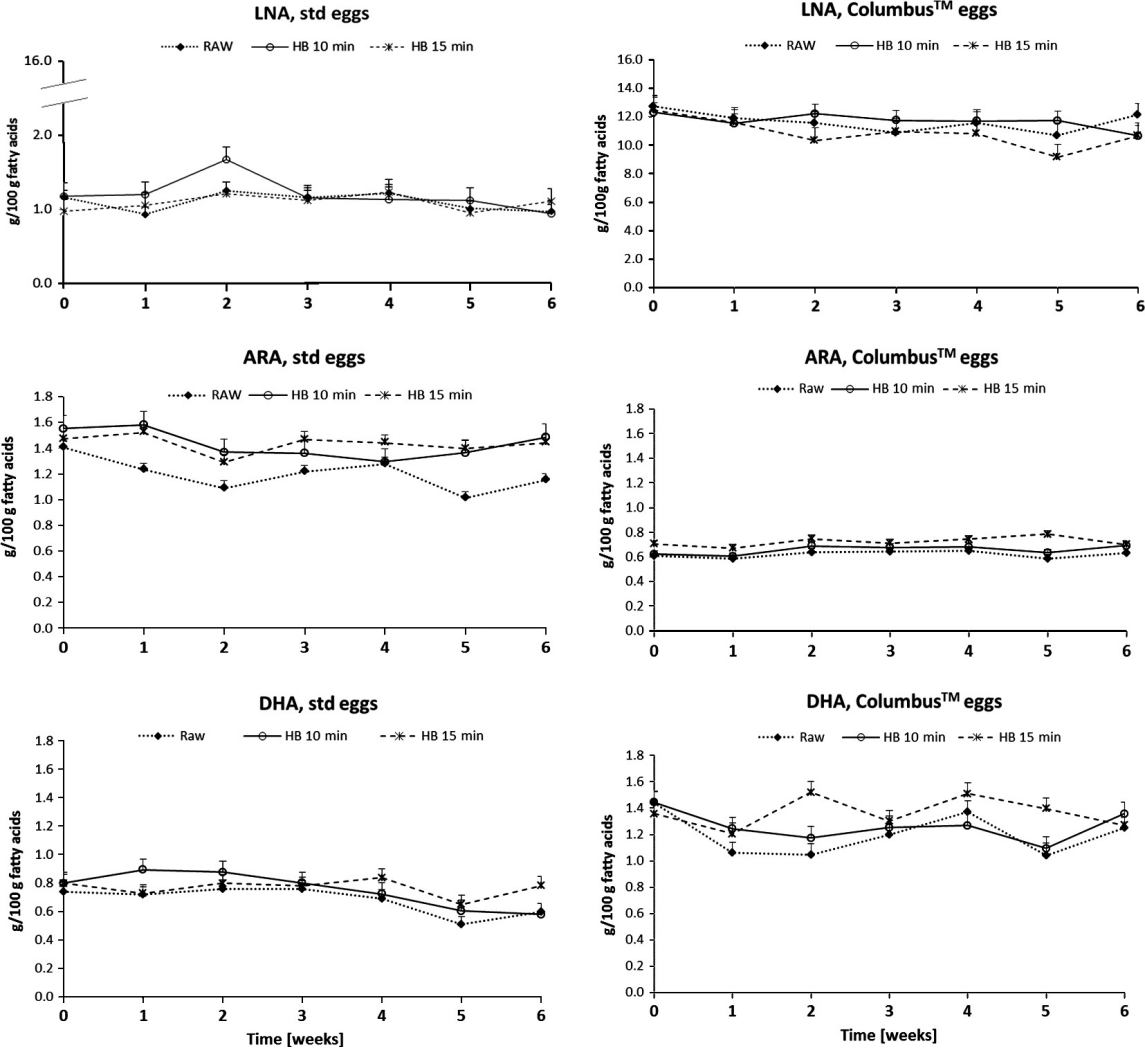
LNA, ARA, and DHA fatty acids content in Columbus™ and standard (std) eggs after storage from 0 to 6 weeks. Eggs were stored raw, hard-boiled 10 min (HB10) and hard-boiled 15 min (HB15). Least Square Means (LSM) ± standard error (SE) of 6 eggs from 4 different batches (day 0) or 4 eggs from one single batch (week 1 to 6) for each storage condition.

Eggs were stored raw, hard-boiled 10 and 15 min, at +4°C and +20°C, during 6 weeks. As no significant difference of the LNA, ARA, and DHA content was observed between the +4°C and +20°C storage conditions, the results from the two different storage temperatures were pooled leading to four repetitions for storage conditions from 1 to 6 weeks. The LNA, ARA, and DHA content in Columbus™ and standard eggs after storage from 0 to 6 weeks are shown in Figure1. After 6 weeks storage, the LNA, ARA, and DHA content measured in Columbus eggs as well as standards eggs showed no significant tendency for a decrease in cooked (hard-boiled) or raw eggs.

Our results are corroborated by Meluzzi et al. (2000) who reported that n-3 rich eggs stored for 28 days at room temperature showed a fatty acids composition similar to that observed in fresh eggs or by Yang et al. (2004) who reported that conjugated linoleic acid (CLA) was stable in eggs during storage for a period of 6 months at 0–4°C. Similarly, Ahn et al. (1999) did not observe the effect of storage on the fatty acid composition when fresh eggs were stored for 49 days at 4°C. Marshall et al. (1994) noticed that storage stability of shell eggs from hens fed 1.5% dietary menhaden oil, a commercial fish oil containing approximately 30% of n-3 fatty acids, is comparable to that from hens fed a no-added fat diet.

On the contrary, a reduction in total n-3 fatty acids of eggs from hens fed with fish oil or olive leaves after 60 days of storage at +4°C was reported (Cherian et al. 2007a,b; Botsoglou et al. 2012c).

As suggested in different studies, the global stability of n-3 fatty acids observed in our experiments of storage and cooking could be explained by the protective effect of *α*- tocopherol. Indeed, *α*-tocopherol is naturally present in raw eggs (Murcia et al. 1999) and is also brought by specific hens feed. According to the inventors of the Columbus feed, the *α*-tocopherol content is around 100 mg kg^−1^ egg in Columbus™ eggs and around 10 mg kg^−1^ egg in standard eggs (Remacle et al. 2001).

### Pork

#### Characterization of raw meat from standard and Columbus ™ pork

The average water content and fat contents were, respectively, 65.8 ± 0.6% and 15.7 ± 0.8% for Columbus™ pork and 67.2 ± 2.7% and 13.9 ± 3.6% for standard pork (*n* = 7), expressed on fresh weight basis. The fatty acid profile of standard and Columbus™ raw meat used as reference in the cooking experiment are shown in Table3. The major difference in the fatty acids composition was coming from the content in n-3 fatty acids, which was of nearly 12% of the total fatty acids in Columbus™ pork, while they were not detectable in standard meat. These omega-3 fatty acids were *α*-linolenic acid (10.4 ± 0.2% LNA of the total fatty acids) and 11,14,17-eicosatrienoic acid (C20:3 n-3) (1.2 ± 0.1% of the total fatty acids), and the n-6/n-3 ratio was close to 1. Longer chain PUFA were not detected neither in Columbus™ meat, neither in standard pork. The nutrition claim “high omega-3 fatty acids” can be used for the Columbus™ pork because it contains more than 6 g omega-3 fatty acids per kg of meat, which is the lower limit for this claim indicated in the Regulation EC no. 1924/2006 (European Parliament and Council Directive No 1924/200620062006, 2006). The PUFA content found in omega-3 pork marketed in Belgium is consistent to what was described in the literature in controlled experiments were pigs were fed with 5% linseed oil containing feed, resulting in LNA content between 8.5% and 9.1% of total fatty acid in pork muscle, with a n-6/n-3 ratio close to 1 (Nurnberg et al. 1999). Other authors reported for the same kind of experiment a LNA content of 7.3% of total fatty acids in pork and a n-6/n-3 ratio of about 2 (Botsoglou et al. 2012d).Some fatty acids were specified as not detected in pork samples used for this study. However, some of these fatty acids were detected and quantified in egg samples used in this manuscript or other meat or food samples used in other studies, in quantities as low as 0.1%. What is more, other studies working with pork did not mention long chain PUFA when presenting the fatty acid profile of “standard” meat (Ramírez et al. 2004, 2005; Paiva-Martins et al. 2009; Cardenia et al. 2011).

**Table 3:** Fatty acid composition (g/100 g) of standard and n-3 enriched (Columbus™) raw pork

	Fatty acid content	
	Columbus™ pork	Standard pork
10:0	ND	ND
12:0	ND	ND
13:0	ND	ND
14:0	**1.3 ± 0.03**	**1.5 ± 0.1**
16:0	**20.0 ± 0.5**	**25.4 ± 0.3**
17:0	ND	ND
18:0	**10.3 ± 0.2**	**13.5 ± 0.2**
20:0	ND	ND
22:0	ND	ND
24:0	ND	ND
16:1 (n-7)	2.2 ± 0.1	2.4 ± 0.1
17:1 (n-7)	ND	ND
18:1 (n-9)	**38.7 ± 0.2**	**46.9 ± 0.5**
18:2 (n-6) (LA)	**15.0 ± 0.5**	**9.7 ± 0.3**
20:2 (n-6)	**1.0 ± 0.04**	**0.7 ± 0.1**
18:3 (n-3) (LNA)	**10.4 ± 0.2**	**ND**
18:3 (n-6)	ND	ND
20:3 (n-3)	**1.2 ± 0.1**	**ND**
18:4 (n-3)	ND	ND
20:4 (n-6) (ARA)	ND	ND
20:5 (n-3) (EPA)	ND	ND
22:5 (n-3) (DPA)	ND	ND
22:6 (n-3) (DHA)	ND	ND
Σ SFA	**31.5 ± 0.7**	**40.3 ± 0.4**
Σ MUFA	**40.9 ± 0.1**	**49.2 ± 0.4**
Σ PUFA	**27.6 ± 0.7**	**10.4 ± 0.3**
n-6 fatty acids	**16.0 ± 0.4**	**10.4 ± 0.3**
n-3 fatty acids	**11.6 ± 0.2**	**–**
n-6/n-3	**1.4 ± 0.02**	**–**

Mean ± standard deviation (SD) of seven different meat samples from two different batches. Significant differences comparing Columbus™ and standard pork are indicated in bold (*P* < 0.05). SFA, saturated fatty acids; MUFA, monounsaturated fatty acids; PUFA, polyunsaturated fatty acids; ND, not detected.

#### Pork cooking experiment

##### Evolution of the core temperature in pork during cooking

The maximum core temperatures recorded at the end of the cooking time (in oven) in the standard and Columbus™ pork were 79.8 ± 1.0°C and 80.1 ± 0.7°C, respectively, (*n* = 4) (data not shown).

#### Fatty acids composition of pork before and after cooking

##### Pork cooking without culinary fat

Standard pork cooked in the oven and in the pan without the use of fat showed no significant change in the content of total SFA, MUFA, or PUFA, compared to raw meat (Table4). For Columbus™ pork, pan frying without fat leaded to a significant decrease in the C20:2(n-6) content but did not significantly affect the n-3 fatty acids (LNA and C20:3) or the sum of PUFA (Table4). Oven cooking (without fat) of the Columbus™ meat showed a larger impact on the fatty acid content of meat, with a significant decrease of LA, LNA, and C20:3(n-3) proportion (14.2% vs. 15.0%, 9.9% vs. 10.4%, and 1.1% vs. 1.2%, in cooked and raw Columbus™ meat, respectively), and a subsequent significant increase of SFA proportion (33.0% vs. 31.6% in cooked and raw Columbus™ meat, respectively) (Table4). This could be due to the high temperature reached in the core of the meat during oven cooking, affecting the PUFA content of Columbus™ meat which is more than two times higher than in standard meat. The same effect was observed for eggs cooked in the oven (Custard and Savoy cake).

**Table 4:** Effect of cooking on the fatty acids composition (g/100 g) of standard and Columbus™ pork

Meat type	Condition	Σ SFA		Σ MUFA		Σ PUFA		n-3		n-6		C18:3(n-3) LNA		C20:3(n-3)		C18:2(n-6) LA		C20:2(n-6)		n-6/n-3	
		LSM	SE	LSM	SE	LSM	SE	LSM	SE	LSM	SE	LSM	SE	LSM	SE	LSM	SE	LSM	SE	LSM	SE
Standard	Raw	40.3	0.3	49.2	0.3	10.4	0.1	ND		10.4	0.1	ND		ND		9.7	0.1	0.7	0.03	**–**	
	Oven (no fat)	40.6	0.3	49.1	0.3	10.3	0.1	ND		10.3	0.1	ND		ND		9.5	0.1	0.7	0.03	**–**	
	Pan (no fat)	40.7	0.4	49.0	0.4	10.3	0.1	ND		10.3	0.1	ND		ND		9.8	0.1	0.6	0.04	**–**	
	Pan (butter)	44.9*	0.4	45.8*	0.4	9.4*	0.1	ND		9.4*	0.1	ND		ND		8.8*	0.1	0.6	0.04	**–**	
	Pan (margarine)	42.8*	0.4	46.5*	0.4	10.8	0.1	ND		10.8	0.1	ND		ND		10.1*	0.1	0.7	0.04	**–**	
	Pan (peanut oil)	38.0*	0.4	50.7*	0.4	11.2*	0.1	ND		11.2*	0.1	ND		ND		10.6*	0.1	0.7	0.04	**–**	
	Pan (sesame oil)	38.0*	0.4	49.0	0.4	13.0*	0.1	ND		13.0*	0.1	ND		ND		12.4*	0.1	0.6	0.04	**–**	
Columbus™	Raw	31.5	0.3	40.9	0.2	27.6	0.2	11.6	0.1	16.0	0.2	10.4	0.1	1.2	0.04	15.0	0.1	1.0	0.04	1.4	0.02
	Oven (no fat)	33.0*	0.3	40.9	0.2	26.1*	0.2	11.0*	0.1	15.1*	0.2	9.9*	0.1	1.1*	0.04	14.2*	0.1	0.9	0.04	1.4	0.02
	Pan (no fat)	31.1	0.5	41.4	0.2	27.5	0.3	11.6	0.2	15.9	0.2	10.4	0.1	1.2	0.1	15.1	0.2	0.8*	0.1	1.4	0.03
	Pan (butter)	37.9*	0.5	38.4*	0.2	23.7*	0.3	9.8*	0.2	13.9*	0.2	8.9*	0.1	0.9*	0.1	13.2*	0.2	0.8*	0.1	1.4	0.03
	Pan (margarine)	35.2*	0.5	39.2*	0.2	25.5*	0.3	10.1*	0.2	15.5	0.2	9.1*	0.1	1.0*	0.1	14.8	0.2	0.7*	0.1	1.5*	0.03
	Pan (peanut oil)	30.4	0.5	44.3*	0.2	25.4*	0.3	9.7*	0.2	15.7	0.2	8.6*	0.1	1.0*	0.1	15.1	0.2	0.7*	0.1	1.6*	0.03
	Pan (sesame oil)	30.2*	0.5	42.1*	0.2	27.6	0.3	9.4*	0.2	18.2*	0.2	8.5*	0.1	1.0*	0.1	17.5*	0.2	0.7*	0.1	1.9*	0.03
Butter^1^		60.7		35.3		4.1		1.2		2.9											
Margarine^1^		42.2		36.3		22.1		3.2		18.9											
Peanut oil^1^		17.2		56.2		26.8		0.2		26.6											
Sesame oil^1^		16.0		42.0		42.0		0.0		42.0											

Least-square means (LSM) and Standard errors (SE) of seven different samples for raw meat (from two different batches), four different samples for oven cooked meat and two different samples for each other cooking condition. Significant differences comparing to the raw condition are indicated by asterisks (^*^) and in bold (*P* < 0.05). SFA, saturated fatty acid**s**; MUFA, monounsaturated fatty acids; PUFA, polyunsaturated fatty acids; n-3, n-3 fatty acids; n-6, n-6 fatty acids; ND, not detected.

1 From Nubel 2009.

##### Pork cooking with culinary fat

As expected, meat cooking including the use of culinary fat induced a larger change in the fatty acid profile of both standard and Columbus™ pork than cooking without the use of fat. Table4 mentions the global fatty acid composition of the culinary fats used in the cooking experiment, according to the Belgian NUBEL food composition table (Nubel 2009). In standard pork cooked with culinary fat, the significant changes in the fatty acid profile are according to the culinary fat used, with an increase of SFA when meat is cooked with butter or margarine and a decrease of PUFA only for meat cooked with butter. When meat is cooked with peanut or sesame oil, a decrease of SFA was observed and an increase of MUFA only for meat cooked with peanut oil (Table4).

In Columbus™ meat, the same observations are valid for SFA and MUFA: increase of SFA and decrease of MUFA when the meat is cooked with butter or margarine and decrease of SFA and increase of MUFA when the meat is cooked with peanut or sesame oil. The PUFA decreased in any case of Columbus™ pork cooked with culinary fat, except for sesame oil (Table4). While looking at the detailed fatty acid profile of cooked Columbus™ pork, it is observed that pan frying with butter, compared to raw meat, resulted in an increase of saturated myristic (C14:0), stearic (C16:0), and palmitic acids (C18:0) from 1.3% to 3.1%, 20.0% to 22.6% for C16:0, and 10.3% to 11.4% for C18:0, respectively (data not shown). A decrease of the percentage of linoleic acid (C18:2n-6) from 15.0% to 13.2% and *α*-linolenic acid (C18:3n-3) from 10.4% to 8.9% was also observed (Table4). Those variations are due to the fact that butter contains high amounts of SFA (more than 50% of total fatty acids) and very few polyunsaturated acids (4.1% only of total fatty acids). When the Columbus™ meat is cooked with vegetable oil, the percentage of oleic acid (C18:1) increases in its fatty acid profile (42.4% with peanut oil and 40.4% with sesame oil vs. 38.7% in raw meat), because of the contribution of oleic acid coming from the oil (data not shown). In all cases of pan-frying experiments with culinary fat, compared to the raw meat, the LNA percentage showed a significant decrease, from 10.4% of total fatty acids in raw meat to 9.1% (cooking with margarine), 8.9% (cooking with butter), and 8.6 and 8.5% (cooking with sesame and peanut oils, respectively) (Table4). This decrease seems not to be due to a loss of LNA, as its percentage remained unchanged when pan frying was performed without fat, but to a dilution effect by the fatty acids coming from the culinary fat, containing very low amount of LNA compared to the Columbus™ meat.

When the meat was cooked with fat, it appeared clearly, that the composition of the culinary fats had a greater influence on its fatty acid profile than the cooking process itself, in both meat types. This was corroborated by the observations of Haak et al. (2007) and Ramírez et al. (2005) who reported that long chain PUFA were not significantly lost by the frying process and that the fatty acids composition of fried pork tended to become similar to that of the culinary fat, as a result of the exchange between culinary fat and meat. The same conclusions were reported by Sioen et al. (2006) for pan-fried fish and by Candela et al. (1998) for deep-fried sardines.

### Pork storage experiment

Columbus™ and standard pork were stored raw for 6 weeks at +4°C or 10 weeks at −20°C. Results obtained at day 0 were compared to results obtained at different times of storage in order to estimate the fatty acid profile evolution. No variation in fatty acids composition was observed after storage for both type of meat. What is more, in Columbus™ meat, the PUFA content remained stable during the whole storage experiment, whatever the temperature of conservation (data not shown).

## Conclusion

A GC–MS method has been developed for the analysis of fatty acids in food matrices and was applied to determine the omega-3 fatty acid profile of Belgian eggs and pork rich in omega-3 fatty acids (Columbus™ eggs or pork), in order to determine to which extent the omega-3 fatty acids resist to storage or cooking. We can conclude that the omega-3 fatty acids remained unchanged in the ready-to-eat product, except for some specific cooking processes (eggs cooked in custard and meat cooked in oven), where a slight statistically significant loss of PUFA in both Columbus™ eggs or pork was observed. As expected, when Columbus™ pork is cooked with culinary fat, its fatty acid profile is modified according to the nature of the fat used.

## References

[b1] AFSSA - Agence Française de Sécurité Sanitaire des Aliments (2003). Acides gras de la famille Oméga 3 et système cardiovasculaire: intérêt nutritionnel et allégations.

[b2] Ahn DU, Sell JL, Jo C, Chamruspollert M, Jeffrey M (1999). Effect of dietary conjugated linoleic acid on the quality characteristics of chicken eggs during refrigerated storage. Poult. Sci.

[b3] Aldai N, Osoro K, Barron LJR, Najera AI (2006). Gas-liquid chromatographic method for analysing complex mixtures of fatty acids including conjugated linoleic acids (cis9trans11 and trans10cis12 isomers) and long-chain (n-3 or n-6) polyunsaturated fatty acids: application to the intramuscular fat of beef meat. J. Chromatogr. A.

[b4] Baucells MD, Crespo N, Barroeta AC, López-Ferrer S, Grashorn MA (2000). Incorporation of different polyunsaturated fatty acids into eggs. Poult. Sci.

[b5] Botsoglou E, Govaris A, Fletouris D, Botsoglou N (2012a). Lipid oxidation of stored eggs enriched with very long chain n−3 fatty acids, as affected by dietary olive leaves (Olea europea L.) or *α*-tocopheryl acetate supplementation. Food Chem.

[b6] Botsoglou E, Govaris A, Fletouris D, Botsoglou N (2012b). Effect of supplementation of the laying hen diet with olive leaves (Olea europeaL.) on lipid oxidation and fatty acid profile of a-linolenic acid enriched eggs during storage. Br. Poult. Sci.

[b7] Botsoglou E, Govaris A, Pexara A, Fletouris D (2012c). Effect of processing and storage on the fatty acid composition of n-3 or n-6 fatty acid-enriched eggs. Int. J. Food Sci. Technol.

[b8] Botsoglou E, Govaris A, Ambrosiadis I, Fletouris D (2012d). Lipid and protein oxidation of *α*-linolenic acid-enriched pork during refrigerated storage as influenced by diet supplementation with olive leaves (Olea europea L.)or *α*-tocopheryl acetate. Meat Sci.

[b9] Candela M, Astiasara′n I, Bello J (1998). Deep-fat frying modifies high-fat fish lipid fraction. J. Agric. Food Chem.

[b10] Cardenia V, Rodriguez-Estrada MT, Cumella F, Sardi L, Della Casa G, Lercker G (2011). Oxidative stability of pork meat lipids as related to high-oleic sunflower oil and vitamin E diet supplementation and storage conditions. Meat Sci.

[b11] Cherian G, Gonzalez D, Ryu KS, Goeger MP (2007a). Long-term feeding of conjugated linoleic acid and fish oil to laying hens: effects on hepatic histopathology, egg quality, and lipid components. J. Appl. Poult. Res.

[b12] Cherian G, Traber MG, Goeger MP, Leonard SW (2007b). Conjugated linoleic acid and fish oil in laying hen diets: effects on egg fatty acids, thiobarbituric acid reactive substances, and tocopherols during storage. Poult. Sci.

[b13] Cortinas L, Galobart J, Barroeta AC, Baucells MD, Grashorn MA (2003). Change in *α*-tocopherol contents, lipid oxidation and fatty acid profile in eggs enriched with linolenic acid or very long-chain *ω*3 polyunsaturated fatty acids after different processing methods. J. Sci. Food Agric.

[b14] Delgado-Lista J, Perez-Martinez P, Lopez-Miranda J, Perez-Jimenez F (2012). Long chain omega-3 fatty acids and cardiovascular disease: a systematic review. Br. J. Nutr.

[b15] European Parliament and Council Directive No 1924/2006 (2006). European Parliament and Council Directive No 1924/2006 of 20 December 2006 on nutrition and health claims made on foods. Official J. Eur. Communities.

[b16] European Parliament and Council Directive No 2002/657/EC (2002). European Parliament and Council Directive No 2002/657/EC of 12 August 2002 implementing Council Directive 96/23 EC concerning the performance of analytical methods and the interpretation of results. Official J. Eur. Communities.

[b17] Ferrier LK, Caston LJ, Leeson S, Squires J, Weaver BJ, Holub BJ (1995). *α*-Linolenic acid- and docosahexaenoic acid-enriched eggs from hens fed flaxseed: Influence on blood lipids and platelet phospholipid fatty acids in humans. Am. J. Clin. Nutr.

[b100] Galobart J, Barroeta AC, Baucells MD, Guardiola F (2001). Lipid Oxidation in Fresh and Spray-Dried Eggs Enriched with ω3 and ω6 Polyunsaturated Fatty Acids During Storage as Affected by Dietary Vitamin E and Canthaxanthin Supplementation. Poult. Sci.

[b18] Haak L, Sioen L, Raes K, Van Camp J, De Smet S (2007). Effect of pan-frying in different culinary fats on the fatty acid profile of pork. Food Chem.

[b19] Halle I, Schöne F (2013). Influence of rapeseed cake, linseed cake and hemp seed cake on laying performance of hens and fatty acid composition of egg yolk. J. für Verbraucherschutz und Lebensmittelsicherheit.

[b20] Harris WS, Mozzaffarian D, Rimm E, Kris-Etherton P, Rudel LL, Appel LJ (2009). Omega-6 fatty acids and risks for cardiovascular disease: a science advisory from the American Heart Association subcommittee of the Council on nutrition, physical activity, and metabolism; council on cardiovascular nursing; and council on epidemiology and prevention. Circulation.

[b21] Hayat Z, Cherian G, Pasha TN, Khattak FM, Jabbar MA (2010). Oxidative stability and lipid components of eggs from flax-fed hens: effect of dietary antioxidants and storage. Poult. Sci.

[b22] Jiang Z, Sim JS (1992). Effects of dietary n−3 fatty acid-enriched chicken eggs on plasma and tissue cholesterol and fatty acid composition of rats. Lipids.

[b23] Lopez-Bote CJ, Sanz Arias R, Rey AI, Castaño A, Isabel B, Thos J (1998). Effect of free-range feeding on n−3 fatty acid and *α*-tocopherol content and oxidative stability of eggs. Anim. Feed Sci. Technol.

[b24] Lovegrove JA, Griffin BA (2013). The acute and long-term effects of dietary fatty acids on vascular function in health and disease. Curr. Opin. Clin. Nutr. Metab. Care.

[b25] Marshall AC, Sams AR, Van Elswyk ME (1994). Oxidative stability and sensory quality of stored eggs from hens fed 1.5% menhaden oil. J. Food Sci.

[b26] Meluzzi A, Sirri F, Manfreda G, Tallarico N, Franchini A (2000). Effects of Dietary Vitamin E on the Quality of Table Eggs Enriched with n-3 Long-Chain Fatty Acids. Poult. Sci.

[b27] Meynier A, Leborgne C, Viau M, Schuck P, Guichardant M, Rannou C (2014). n-3 fatty acid enriched eggs and production of egg yolk powders: an increased risk of lipid oxidation?. Food Chem.

[b28] Murcia MA, Martinez-Tome M, del Cerro I, Sotillo F, Ramirez A (1999). Proximate composition and vitamin E levels in egg yolk: losses by cooking in a microwave oven. J. Sci. Food Agric.

[b29] Nubel (2009). Belgian food composition table (Table belge de composition des aliments).

[b30] Nurnberg K, Kuchenmeister U, Nurnberg G, Ender K, Hackl W (1999). Influence of exogenous application of n-3 fatty acids on meat quality, lipid composition, and oxidative stability in pigs. Arch. Animal Nutr.

[b31] Oeffner SP, Qu Y, Just J, Quezada N, Ramsing E, Keller M (2013). Effect of flaxseed supplementation rate and processing on the production, fatty acid profile, and texture of milk, butter, and cheese. J. Dairy Sci.

[b32] Paiva-Martins F, Barbosa S, Pinheiro V, Mourão JL, Outor-Monteiro D (2009). The effect of olive leaves supplementation on the feed digestibility, growth performances of pigs and quality of pork meat. Meat Sci.

[b33] Priego-Capote F, Ruiz-Jiménez J, GarcÍa-Olmo J, Luque de Castro MD (2004). Fast method for the determination of total fat and trans fatty-acids content in bakery products based on microwave-assisted Soxhlet extraction and medium infrared spectroscopy detection. Anal. Chim. Acta.

[b34] Priego-Capote F, Ruiz-Jiménez J, Luque de Castro MD (2007). Identification and quantification of trans fatty acids in bakery products by gas chromatography–mass spectrometry after focused microwave Soxhlet extraction. Food Chem.

[b35] Raes K, De Smet S, Demeyer D (2004). Effect of dietary fatty acids on incorporation of long chain polyunsaturated fatty acids and conjugated linoleic acid in lamb, beef and pork meat: a review. Anim. Feed Sci. Technol.

[b36] Ramírez MR, Estévez M, Morcuende D, Cava R (2004). Effect of the Type of Frying Culinary Fat on Volatile Compounds Isolated in Fried Pork Loin Chops by Using SPME-GC-MS. J. Agr. Food chem.

[b37] Ramírez M, Morcuende D, Estévez M, Cava López R (2005). Fatty acid profiles of intramuscular fat from pork loin chops fried in different culinary fats following refrigerated storage. Food Chem.

[b38] Remacle C, Lignian J, Erpicum T, De Meester F, Coucke L, Sim J (2001).

[b39] Ren Y, Perez TI, Zuidhof MJ, Renema RA, Wu J (2013). Oxidative stability of omega-3 polyunsaturated fatty acids enriched eggs. J. Agric. Food Chem.

[b40] Ruiz-Rodriguez A, Reglero G, Ibanez E (2010). Recent trends in the advanced analysis of bioactive fatty acids. J. Pharm. Biomed. Anal.

[b41] Samman S, Kung FP, Carter LM, Foster MJ, Ahmad ZI, Phuyal JL (2009). Fatty acid composition of certified organic, conventional and omega-3 eggs. Food Chem.

[b42] Simopoulos AP (2008). The importance of the omega-6/omega-3 fatty acid ratio in cardiovascular disease and other chronic diseases. Exp. Biol. Med.

[b43] Sioen I, Haak L, Raes K, Hermans C, De Henauw S, De Smet S (2006). Effects of pan-frying in margarine and olive oil on the fatty acid composition of cod and salmon. Food Chem.

[b44] Van Elswyk ME, Sams AR, Hargis PS (1992). Composition, functionality, and sensory evaluation of eggs from hens fed dietary Menhaden oil. J. Food Sci.

[b45] Virot M, Tomao V, Colnagui G, Visinoni F, Chemat F (2007). New microwave-integrated Soxhlet extraction: an advantageous tool for the extraction of lipids from food products. J. Chromatogr. A.

[b46] Virot M, Tomao V, Ginies C, Visinoni F, Chemat F (2008). Microwave-integrated extraction of total fats and oils. J. Chromatogr. A.

[b47] Wood JD, Richardson RI, Nute GR, Fisher AV, Campo MM, Kasapidou E (2004). Effects of fatty acids on meat quality: a review. Meat Sci.

[b48] Yang L, Cao Y, Chen ZY (2004). Stability of conjugated linoleic acid isomers in egg yolk lipids during frying. Food Chem.

